# *Anopheles* salivary gland proteomes from major malaria vectors

**DOI:** 10.1186/1471-2164-13-614

**Published:** 2012-11-13

**Authors:** Albin Fontaine, Thierry Fusaï, Sébastien Briolant, Sylvain Buffet, Claude Villard, Emilie Baudelet, Mathieu Pophillat, Samuel Granjeaud, Christophe Rogier, Lionel Almeras

**Affiliations:** 1 Unité de Parasitologie – UMR6236, URMITE – IFR48, Antenne Marseille de l’Institut de Recherche Biomédicale des Armées (IRBA), BP 60109, Marseille Cedex 07, 13 262, France; 2 Unité de Recherche sur les Maladies Infectieuses et Tropicales Emergentes (URMITE), UMR 6236, Faculté de médecine, 27 Bd Jean Moulin, Marseille cedex 5, 13385, France; 3 Plateau Protéomique Timone, FRE CNRS 2737 CISMET, université Aix-Marseille II, 27 Bd Jean Moulin, Marseille cedex 5, 13385, France; 4 Plateforme de Spectrométrie de Masse et de Protéomique, Centre de Recherche en Cancérologie de Marseille, U1068, INSERM/Institut Paoli-Calmettes, 27 Bd Leï Roure, Marseille Cedex 9, BP 30059, 13273, France; 5 Centre d'Immunologie de Marseille Luminy (CIML), Institut National de la Santé et de la Recherche Médicale, Centre National de la Recherche Scientifique, Université de la Méditerranée, Parc Scientifique de Luminy, Case 906, Marseille Cedex 9, Marseille, 13288, France; 6 TAGC INSERM ERM 206, Parc Scientifique de Luminy, Case 928, Marseille Cedex 9, 13288, France; 7 Institut Pasteur de Madagascar, B.P. 1274, Antananarivo, 101, Madagascar

**Keywords:** *Anopheles*, Salivary proteins, Sequence alignment, Biomarkers, Malaria vectors, Protein diversity

## Abstract

**Background:**

Antibody responses against *Anopheles* salivary proteins can indicate individual exposure to bites of malaria vectors. The extent to which these salivary proteins are species-specific is not entirely resolved. Thus, a better knowledge of the diversity among salivary protein repertoires from various malaria vector species is necessary to select relevant genus-, subgenus- and/or species-specific salivary antigens. Such antigens could be used for quantitative (mosquito density) and qualitative (mosquito species) immunological evaluation of malaria vectors/host contact. In this study, salivary gland protein repertoires (sialomes) from several *Anopheles* species were compared using *in silico* analysis and proteomics. The antigenic diversity of salivary gland proteins among different *Anopheles* species was also examined.

**Results:**

*In silico* analysis of secreted salivary gland protein sequences retrieved from an NCBInr database of six *Anopheles* species belonging to the *Cellia* subgenus (*An. gambiae*, *An. arabiensis*, *An. stephensi* and *An. funestus*) and *Nyssorhynchus* subgenus (*An. albimanus* and *An. darlingi*) displayed a higher degree of similarity compared to salivary proteins from closely related *Anopheles* species. Additionally, computational hierarchical clustering allowed identification of genus-, subgenus- and species-specific salivary proteins. Proteomic and immunoblot analyses performed on salivary gland extracts from four *Anopheles* species (*An. gambiae, An. arabiensis*, *An. stephensi* and *An. albimanus*) indicated that heterogeneity of the salivary proteome and antigenic proteins was lower among closely related anopheline species and increased with phylogenetic distance.

**Conclusion:**

This is the first report on the diversity of the salivary protein repertoire among species from the *Anopheles* genus at the protein level. This work demonstrates that a molecular diversity is exhibited among salivary proteins from closely related species despite their common pharmacological activities. The involvement of these proteins as antigenic candidates for genus-, subgenus- or species-specific immunological evaluation of individual exposure to *Anopheles* bites is discussed.

## Background

Several mosquito species of the *Anopheles* genus are vectors of *Plasmodium* parasites, the causal agents of malaria. This major vector-borne disease affects around 216 million individuals annually and leads to more than 600,000 deaths, mainly in tropical and sub-tropical countries [1]. Among approximately 470 *Anopheles* species indexed worldwide [2,3], 34 species found in different regions around the world are considered to be the main vectors of the four *Plasmodium* parasite species (*P. falciparum*, *P. vivax*, *P. ovale* and *P. malariae*) responsible for human malaria [4]. *An. funestus* and two sister-species of the *An. gambiae sensu lato (s.l.)* species complex (*i.e.*, *An. gambiae* and *An. arabiensis*) are primary vectors of *P. falciparum* malaria in sub-Saharan Africa [5], where 80% of malaria mortality and morbidity occur [6]. Among other anopheline vectors of medical importance, *An. stephensi* plays a prominent role in urban malaria transmission in the Indo-Pakistan subcontinent [7,8], and both *An. albimanus* and *An. darlingi* are primary vectors of malaria in Central America and various areas of South America [9-11].

In the absence of a licensed malaria vaccine [12,13] and while *Plasmodium* drug resistance spreads across the world [14], vector control is still the most effective method to protect people from arthropod-borne diseases [15]. Prevention of arthropod infective bites can be achieved by personal protective measures and vector control strategies [15,16]. The evaluation of the effectiveness of these anti-vectorial measures is essentially based on entomological methods such as measuring the density of a mosquito species relative to human density [17]. Catching human landing mosquitoes is currently the most reliable method to estimate host/vector contact [18,19]. Entomological parameters are also a component of numerous indices used to monitor malaria transmission [17,20,21]. However, entomological methods for evaluating the risk of malaria transmission are mainly applicable to the population level and are poorly efficient at evaluating heterogeneity in exposure to vector bites among individuals due to considerable variation of exposure within small geographic areas [22,23] or heterogeneity in socioeconomic and demographic factors (*e.g.* age of humans). Furthermore, the human landing catch method is labour-intensive, has budgetary and logistical constraints and is hampered by ethical limitations with the deliberate exposure of individuals to mosquito-borne pathogens. Thus, alternative cost-effective and convenient methods need to be developed to assess human exposure to malaria vectors. During their blood meal, mosquitoes inject saliva into the host’s skin. This saliva contains a cocktail of active components that counteract host haemostasis and modulate immune responses to ensure blood meal success [24,25]. Secreted salivary proteins of mosquitoes have been reported to elicit antibody responses in people living in endemic areas [26-29] and among travellers transiently exposed to vector bites in tropical areas [30]. These antibody responses were described as being short lived and linked to the level of exposure [28,30,31], highlighting the potential use of these responses to arthropod saliva antigens as immunological markers to evaluate individual exposure to arthropod bites [32] or assess the impact of vector control interventions [33]. Several studies demonstrated the presence of cross-reactive antibody responses against salivary proteins from different hematophagous arthropod species [34-38]. This cross-reactivity was attributed to the existence of antigens shared among different vector species [39]. Species-specific antibody responses against salivary proteins from arthropods have repeatedly been reported [40-42]. Thus, variable levels of homology between salivary protein sequences from different hematophagous arthropods could determine their specificity or cross-recognition [43]. Recently, availability of the genome sequence of several arthropods of major health importance [44,45], combined with transcriptomic and proteomic analyses of their salivary gland extracts (SGEs) [46-54] have provided new insight into the diversity of salivary molecules among various hematophagous arthropods [55]. These studies revealed a number of secreted protein families, potentially involved in haematophagy or sugar digestion, that are ubiquitous in the Nematocera suborder. Completion of *Culex quinquefasciatus*, *Aedes aegypti* and *Anopheles gambiae* genome sequences also led to the discovery of genus-specific salivary proteins [55]. For *Plasmodium spp.* vectors, salivary gland transcriptomes and proteomes of *An. gambiae* (*Cellia sb.*) [47,56], *An. stephensi* (*Cellia sb.*) [52], *An. funestus* (*Cellia sb.*) [57] and *An. darlingi* (*Nyssorhynchus sb.*) [48,49] have been examined to date, providing a thorough description of the salivary protein repertoire from these main malaria vectors throughout the world. Notably, secreted salivary proteins were found to be more divergent than housekeeping proteins, indicating a rapid evolution of these proteins within the *Anopheles* genus [48,49,52]. However, sialome diversity among these different anopheline species is poorly documented at the molecular and antigenic levels.

Six *Anopheles* species were selected according to their major role in human malaria parasite transmission (*i.e.*, *An. gambiae*, *An. arabiensis*, *An. stephensi*, *An. funestus*, *An. albimanus* and *An. darlingi*). Selection of these species was also motivated by the various degrees of phylogenetic relationships among them (*i.e.*, species from the same genus belonging to different subgenera and species complex) and access to their sialomes via salivary gland dissection or through protein sequences obtained by conceptual translation of mRNA sequences previously identified in sialotranscriptomic studies. Importantly, few protein sequences are available for *An. arabiensis* and *An. albimanus* due to the lack of sialotranscriptomic studies conducted on these species. Recently, assembly of transcriptional sequences derived from several body tissues including salivary glands of adult female *An. albimanus* was performed [58]. However, merging the sequence data from the different tissues into a single assembly did not allow clustering secreted salivary protein sequences from the others mosquito body parts, thereby restricting the number of available salivary protein sequences for this species. Salivary gland protein (SGP) repertoires of these different *Anopheles* species were compared in the present study using *in silico* analysis and proteomics approaches to assess their diversity at the molecular and protein levels. Conceptual secreted salivary gland protein sequences retrieved from an NCBInr database of six *Anopheles* species were clustered according to their level of amino acid identity to identify both conserved protein families throughout the *Anopheles* genus and sub-genus- or species- specific salivary proteins. Proteins contained in salivary gland extracts from four *Anopheles* species were separated by 1-D SDS-PAGE and identified by tandem mass spectrometry (MS/MS). The antigenic diversity of SGPs was also examined by immunoblot analysis. Collectively, these data represent the first report of genus-, subgenus- and species-specific *Anopheles* secreted salivary proteins. These proteins could be used for immunological evaluation of the exposure to *Anopheles* bites.

## Results

### Phylogenetic relationships between selected anopheline species

**Figure 1:**
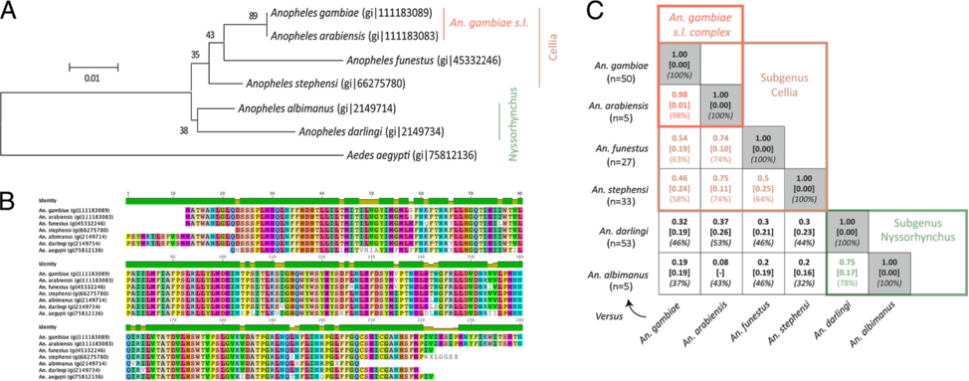
**Salivary protein sequence comparisons among six anopheline species.** (**A**) Phylogenetic relationships among six *Anopheles* species using the cytochrome oxidase subunit II (COII) protein sequences. Evolutionary analyses were conducted in MEGA5 [62]. The *Aedes aegypti* sequence was taken as an outgroup. The tree is drawn to scale with branch lengths in the same units as those of the evolutionary distances used to infer the phylogenetic tree. (**B**) Clustal alignment. The numbers into brackets in the sequence titles indicate the NCBI accession number. The level of sequence identity is graphically represented above sequences alignment. (**C**) Average normalised BLAST scores ± standard deviations (numbers in bold into square brackets) and percentage identities (numbers in italic into brackets) between local alignments of secreted salivary proteins pertaining to sialomes from different *Anopheles* species. Pairwise protein-protein sequence comparisons were performed using “BLAST 2 Sequences” [63] (*q.v.* Additional file 1). This analysis of divergence among secreted salivary protein repertoires was carried out using all protein sequences from each *Anopheles* species matching at least one other salivary protein in another species at 40% identity (*q.v.* Additional file 2). The number of secreted salivary proteins used in each species is indicated into brackets.

Six *Anopheles* species (*An. gambiae*, *An. arabiensis*, *An. stephensi*, *An. funestus*, *An. albimanus* and *An. darlingi*) were selected based on their significance as major vectors of human malaria in different parts of the world. Despite the availability of an *An. albimanus* transcriptome, protein sequences specifically matching secreted salivary gland proteins could not be identified in the whole body dataset [58]. This results from the hybrid nature of the transcriptome data from different tissues of adult female *An. albimanus* into a single transcriptome dataset, from which salivary gland-specific transcriptomic data have been excluded due to their low representation compared to other tissues. However, the sialome of four of the species (*An. gambiae*, *An. stephensi*, *An. funestus* and *An. darlingi*) has been characterised by high-throughput sialotranscriptomic studies [47,49,52,57,59]. These six *Anopheles* species were gathered in phylogenetically meaningful groups by analysing the degree of similarity of their cytochrome oxidase subunit II (COII) protein sequences (Figure 1A, 1B) [60,61]. Alignment of the six protein sequences shows 100% identity between *An. gambiae* and *An. arabiensis* (Figure 1B). The orthologous COII protein sequences from the others *Anopheles* species are more divergent with 97.1%, 95.2%, 97% and 96.6% identity for *An. funestus*, *An. stephensi*, *An. albimanus* and *An. darlingi*, respectively. A bootstrap consensus tree inferred from 10,000 replicates showed that the six *Anopheles* species are divided into the following two major groups based on their taxonomic classification: (i) a clade formed by *An. gambiae*, *An. arabiensis*, *An. funestus* and *An. stephensi*, which are all members of the *Cellia* subgenus; and (ii) a clade including *An. albimanus* and *An. darlingi*, which are two neotropical species belonging to the *Nyssorhynchus* subgenus. The large *Cellia* clade encompasses *An. gambiae* and *An. arabiensis*, which are two morphologically indistinguishable sibling species from the *An. gambiae s.l.* species complex. This phylogenetic analysis also indicated larger genetic distance between the *An. gambiae s.l.* (subgenus *Cellia*, Pyretophorus Series), *An. funestus* (subgenus *Cellia* Myzomyia Series) and *An. stephensi* (subgenus *Cellia*, Neocellia Series) species.

### Comparison of secreted salivary protein sequences retrieved from public databases

A total of 401 salivary gland protein sequences from the six *Anopheles* species were retrieved from public databases according to their annotation. Focusing on proteins potentially injected into the human host during mosquito blood feeding, protein sequences were sorted based on signal peptide predictions [64,65]. A total of 272 out of these 401 salivary proteins, heterogeneously distributed among the six *Anopheles* species (*i.e.,* 71, 5, 44, 5, 117 and 30 protein sequences for *An. gambiae*, *An. arabiensis*, *An. stephensi*, *An. albimanus*, *An. darlingi* and *An. funestus*, respectively), were predicted to harbor a secretory-signal peptide following submission to SignalP server 3.0 and were thus retained for further analysis. A pairwise protein-protein sequence comparison was first performed on these selected SGPs using “BLAST 2 Sequences” [63] to find regions of local similarity between sequences from different *Anopheles* taxa. Briefly, secreted salivary protein sequences from each *Anopheles* species were gathered into six different databases in accordance with their species affiliation. Each protein from a database was then searched against all proteins from other database in a pairwise fashion. BLAST E-values were used as a parsing criterion in order to select best matches between two different salivary protein repertoires. In order to perform comparative analysis between salivary proteins repertoires, Raw BLAST score obtained from the match of a query protein sequence with a targeted protein sequence were divided against raw self-BLAST score from the match of the query protein sequence to itself to obtain normalised BLAST scores (or BLAST Score Ratio). Normalised BLAST scores range from 0 (no BLAST match) to 1 (perfect BLAST match between two salivary proteins) [66,67]. The average normalised BLAST scores and average percentage identity between local alignments that estimate the degree of homology between salivary protein sequences from each pair of *Anopheles* species are presented in Figure 1C and Additional file 1. Only protein sequences from each *Anopheles* species matching at least one other salivary protein in another species at 40% identity (*q.v.* Additional file 2) were considered in this analysis, representing 50, 5, 33, 5, 53 and 27 protein sequences for *An. gambiae*, *An. arabiensis*, *An. stephensi*, *An. albimanus*, *An. darlingi* and *An. funestus*, respectively. The average normalised BLAST score between salivary protein sequences from *An. gambiae* and *An. arabiensis* was 0.98 ± 0.01 (mean ± SD). Based on the same criteria, salivary protein sequences from both species from the *Nyssorhynchus* subgenus had normalised BLAST scores of 0.75 ± 0.17 and sequences from species belonging to the *Cellia* subgenus had normalised BLAST scores larger than 0.45. Lower normalised BLAST scores were observed when comparing salivary protein sequences from species belonging to *Cellia* with those of the *Nyssorhynchus* subgenera (all normalised BLAST scores were inferior to 0.40) (Figure 1C, Additional file 1). Thus, secreted salivary protein sequence similarities were the highest among closely related anopheline species and decreased with increasing phylogenetic distance (Figure 1A, B).

### Hierarchical clustering of secreted protein sequences

**Figure 2:**
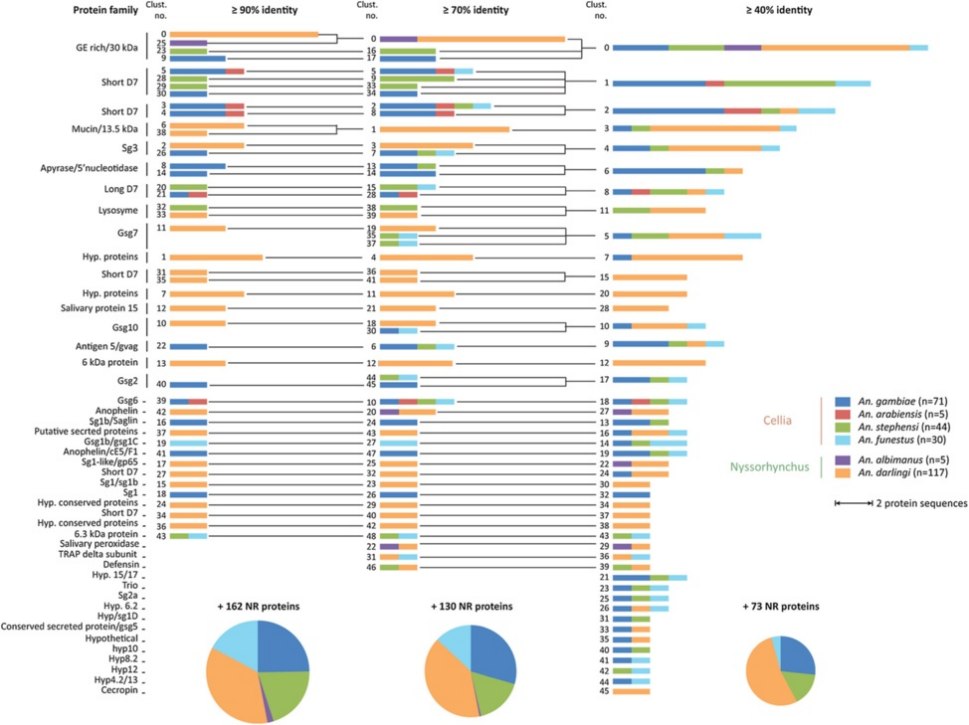
**Hierarchical clustering of secreted salivary protein sequences from *Anopheles*.** Three clustering steps were performed sequentially at different similarity thresholds (≥ 90%, ≥ 70% and ≥ 40% identity), producing a hierarchical structure. The repartition of proteins from the *Anopheles* species into clusters of more than 2 protein sequences are proportionally represented by stacked bars and non-redundant (NR) protein sequences (*i.e*., sequences that were not clustered with other sequences over a specified similarity threshold) by pie charts. The cluster numbers indicated on the left side of the stacked bars correspond to protein clusters listed in Additional file 2. A total of 71, 5, 44, 30, 5 and 117 secreted salivary protein sequences were recovered from the NCBInr online database for *An. gambiae*, *An. arabiensis*, *An. stephensi*, *An. funestus*, *An. albimanus* and *An. darlingi*, respectively. The correspondence between the number of proteins in a cluster and length of stacked bars is indicated as well as the correspondence between the colours and each *Anopheles* species.

Hierarchical clustering of the salivary protein sequences was performed to determine paralogous (*i.e.*, homologous intra-species protein derived from a gene duplication event) and orthologous (*i.e.*, homologous inter-species protein derived from a speciation event) salivary proteins and their degrees of similarity among the six *Anopheles* species. Three clustering steps using the CD-HIT program [68] were sequentially performed at different similarity thresholds based on full-length sequences (≥ 90%, ≥ 70% and ≥ 40% identity). This agglomerative hierarchical clustering approach was used to maximise the quality of clustering [69] and produce a tree-like structure (Figure 2) to assess the level of homology among the proteins. Among the 272 secreted salivary proteins that were retrieved, the first clustering step (≥ 90% identity threshold) led to the determination of 162 (60%) non-redundant (NR) protein sequences (*i.e.*, sequences that did not cluster with other sequences over a specified identity threshold) and 44 clusters composed of at least two protein sequences (mean number of proteins per cluster ± 95% confidence interval (CI) of the mean, 2.63 ± 0.36). These 44 clusters were almost exclusively composed of paralogous sequences with the exception of five *An. arabiensis* protein sequences, which all clustered with *An. gambiae* sequences (clusters 3, 4, 5, 21, 39) and cluster 43 composed of *An. stephensi* and *An. funestus* 6.3 kDa proteins (Figure 2, Additional file 2). The second clustering steps (≥ 70% identity threshold) identified 130 (48%) NR protein sequences and 49 clusters (2.94 ± 0.46). Among these 49 clusters, 19 consisted of orthologous protein sequences. The vast majority of salivary protein sequences from species belonging to the *Nyssorhynchus* subgenus did not cluster with those from the *Cellia* subgenus at this step (Figure 2A and Additional file 2). The last clustering steps (≥ 40% identity threshold) resulted in 73 (27%) NR protein sequences and 46 clusters (4.37 ± 1). Among these clusters, 36 were composed of orthologous protein sequences, half of which (18 out of 36) consisted of sequences belonging to both *Nyssorhynchus* and *Cellia* subgenera. These orthologous sequences belong to several protein families, including the apyrase/5’nucleotidase, antigen 5/gvag, GE-rich/30 kDa, long and short form D7, mucin/13.5 kDa, SG3, SG7, SG10 or hypothetical 6.2 kDa protein families. Among the NR sequences, 12 out of 19 (63%), 18 out of 38 (47%), 5 out of 11 (45%) and 1 out of 3 (33%) have no homologs in sialomes from other blood feeding arthropods, concerning *An. gambiae*, *An. darling*i, *An. stephensi* and *An. funestus*, respectively. A majority of these species-specific secreted salivary proteins have low molecular weights (Additional file 2).

### Analysis of salivary gland protein repertoires from four *Anopheles* species

**Figure 3:**
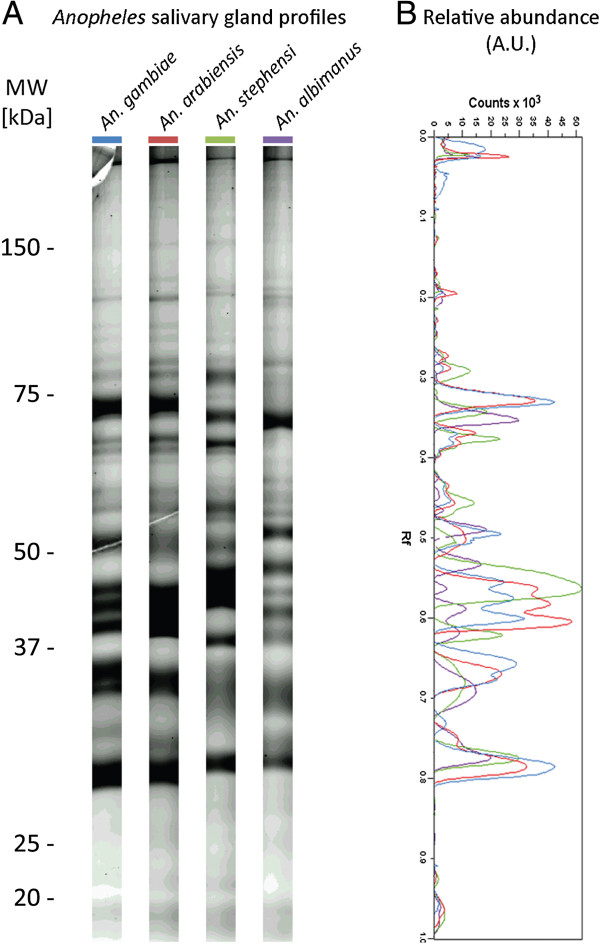
**Salivary gland protein profiles among four *Anopheles *species.** Salivary gland proteins collected from *An. gambiae*, *An. arabiensis*, *An. stephensi* and *An. albimanus* were separated on 12% SDS-PAGE gels and stained with Sypro Ruby. The *Anopheles* species, corresponding to each protein track, are indicated at the top of the gel. Standard molecular masses are indicated on the left side. (**B**) Densitometric protein profiles of salivary gland proteins from the four *Anopheles* species are presented. Species are indicated by the same colour at the top of each immunoblot profile. MW, molecular weight; kDa, kiloDalton; A.U., arbitrary units; Rf, relative front of migration.

The salivary protein sequence repertoires available from public databases for both *An. arabiensis* and *An. albimanus* species are largely incomplete, and the vast majority of *Anopheles* protein sequences are inferred from transcriptomic or genomic sequence analyses. Thus, a proteomic analysis was performed to confirm the existence of predicted secreted proteins and evaluate the sequence diversity observed by *in silico* analysis at the protein level. Access to SGEs could only be achieved for four of the six *Anopheles* species by dissecting wild mosquitoes (*An. arabiensis*) or mosquitoes reared in laboratories (*An. gambiae*, *An. stephensi* and *An. albimanus*). Nonetheless, these four selected species are a representative sample of the *Anopheles* taxonomic diversity at the subgenus, species complex and species levels. SGPs of the four *Anopheles* species were separated by SDS-PAGE (Figure 3A). Despite slight quantitative variations (*i.e.,* band intensities), superimposition of densitometric protein profiles indicated a high similarity between the *An. gambiae* and *An. arabiensis* species belonging to the *An. gambiae s.l.* species complex (Figure 3B). Conversely, SGP profiles of *An. stephensi* (*Cellia* sb.) and *An. albimanus* (*Nyssorhynchus* sb.) differed and were highly distinct from *An. gambiae s.l.* profiles at the qualitative (*i.e.*, molecular weights of the bands) and quantitative (*i.e.*, band intensities) levels.

**Figure 4:**
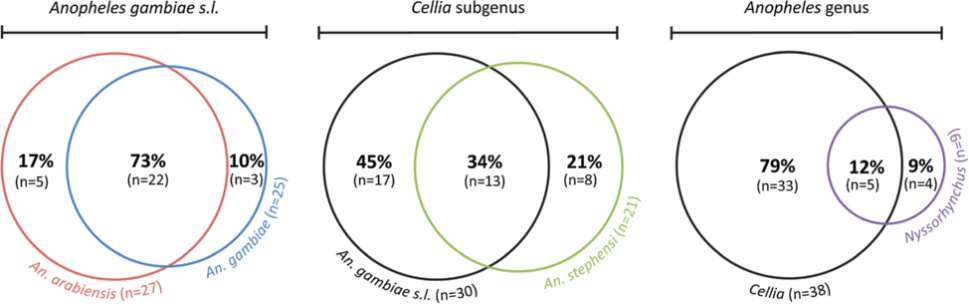
**Venn diagrams indicating the amount of secreted salivary proteins identified in four *Anopheles *species.** The amount of putative secreted proteins identified by MS in *An. gambiae*, *An. arabiensis*, *An. stephensi* and *An. albimanus* SGEs was represented at each taxonomic level (*q.v.*, Figure 1A, 1B). The percentage of proteins identified is indicated in bold with the corresponding number of protein in brackets.

To improve estimates of protein diversity, SGP repertoires from these four mosquito species were identified as previously described [54,70]. Briefly, each gel loading track was cut into several segments covering the entire protein profile and proteins from gel pieces were identified by MS/MS. As scarce protein sequences are available in protein database for some of the *Anopheles* species under study, the MS/MS spectra were searched against a non-redundant protein database including protein sequences from *An. gambiae, An. arabiensis, An. stephensi* and *An. albimanus* together. This strategy was implemented to identify homologous proteins among *Anopheles* species based on their peptides similarities. A total of 41, 44, 40 and 16 proteins were identified in SGEs from *An. gambiae*, *An. arabiensis*, *An. stephensi* and *An. albimanus*, respectively, representing a total of 77 unique proteins for all species (Additional file 3). Among these 77 unique proteins, 25, 27, 21 and 9 were identified as putative secreted proteins in the SGEs of *An. gambiae*, *An. arabiensis*, *An. stephensi* and *An. albimanus*, respectively, totalling 42 unique putative secreted proteins for all species (Additional file 4). Only 26, 2, 11 and 3 salivary proteins were identified in *An. gambiae*, *An. arabiensis*, *An. stephensi* and *An. albimanus*, respectively. The majority of these proteins (18/42) were identified in the *An. gambiae strain PEST* by homology. Protein members from the apyrase and 30 kDa/GE-rich/anti-platelet families were identified in all *Anopheles* species. A total of 18 secreted proteins were only identified in unique *Anopheles* species. The number of common proteins among the *Anopheles* species was highest among closely related anopheline species (*i.e.*, 73% among *An. gambiae* and *An. arabiensis*) and decreased with increasing phylogenetic distance (*i.e.*, 34% of proteins were identified in common among species from the *Cellia* subgenus and 12% of proteins were common among the four *Anopheles* species (Figure 4).

### Antigenic heterogeneity of *Anopheles* salivary gland proteins

**Figure 5:**
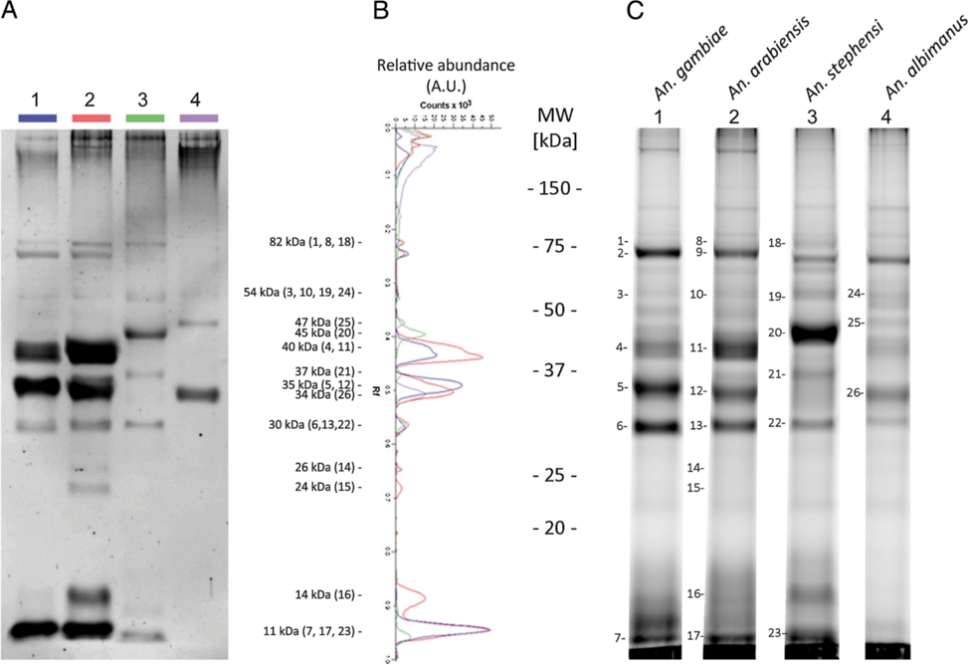
**Singularity of IgG immune profiles among the *Anopheles *species.** Fifteen micrograms of salivary gland extracts from *An. gambiae* (1), *An. arabiensis* (2), *An. stephensi* (3) and *An. albimanus* (4) labelled with Cyanine 5, were loaded and separated by 12% SDS-PAGE. (**A**) IgG immune profiles from pooled sera from 5 Senegalese individuals exposed to *An. gambiae s.l.* and *An. funestus* were tested by immunoblotting experiments. (**B**) Normalised densitometric IgG profiles were represented for the four *Anopheles* species. Species are indicated by the same colour at the top of each immunoblot profile. Molecular weights of the antigenic bands are indicated and corresponding gel bands are presented into brackets. (**C**) Protein profiles of whole protein present in SGEs from each mosquito species were scanned at the Cy5 wavelength before blotting. The numbers correspond to antigenic protein bands excised for mass spectrometry identification (Additional file 5). MW, molecular weight; kDa, kiloDalton; Rf, relative front of migration; A.U., Arbitrary Unit.

**Figure 6:**
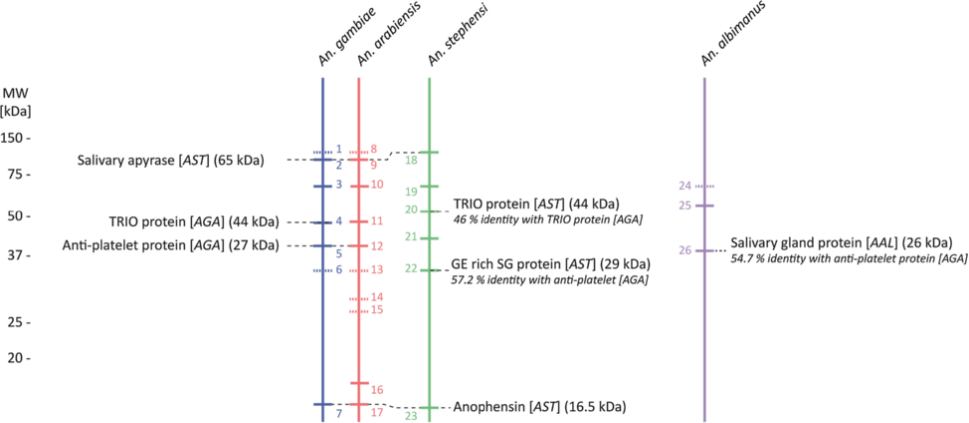
**Schematic representation of the identified antigenic protein bands in salivary gland extracts from four *Anopheles *species.** Secreted salivary proteins identified in antigenic bands (*q.v.,* Additional file 5) are indicated with their corresponding species into squared brackets and their molecular weights. No protein was identified in antigenic bands represented by dotted lines. The percentage identity between two protein sequences was either retrieved from the *in silico* analysis (Additional file 2) or from analysis of “BLAST 2 Sequences”. Coloured numbers correspond to protein bands from the gel from the Figure 5C. MW, molecular weight; AGA, *An. gambiae*; AAR. *An. arabiensis*, AST, *An. stephensi*; AAL, *An. albimanus* .

Protein sequence diversity observed in *in silico* and proteomic analyses among the four *Anopheles* species was also tested at the antigenic level. Using a pool of sera from 5 Senegalese individuals exposed mainly to *An. gambiae s.l.* and *An. funestus*[71], a comparison of IgG antibody responses against SGEs from *An. gambiae, An. arabiensis, An. stephensi* and *An. albimanus* species was performed by immunoblot analysis. Several antigenic bands were observed in SGEs of all anopheline species, with a total of 7, 10, 6 and 3 antigenic bands detected in *An. gambiae, An. arabiensis, An. stephensi* and *An. albimanus*, respectively (Figure 5A). The pooled sera exhibited high reactivity with three antigenic bands at 40, 35 and 11 kDa in SGEs from *An. gambiae s.l.,* and all other antigenic bands detected in SGEs from *An. gambiae* were also found in those from *An. arabiensis.* However, 3 antigenic bands at 26, 24 and 14 kDa were exclusively observed in *An. arabiensis* SGEs (Figure 5A and 5B). Three antigenic bands with molecular weights of 82, 30 and 11 kDa were recognised in SGEs from species belonging to the *Cellia* subgenus (*i.e.*, *An. stephensi* and *An. gambiae s.l.*). However, the antigenic band with low molecular weight (11 kDa) was 7.5-fold less intense in *An. stephensi* compared to those of the same molecular weight in *An. gambiae s.l.* With the same molecular weight criteria, two antigenic bands (45 and 37 kDa) were only detected in the *An. stephensi* SGE antigenic profile. In *An. albimanus* SGEs, three antigenic bands were detected with molecular weights of 54, 47 and 34 kDa. Among them, the more intense antigenic band (34 kDa) was 2.8-fold less intense than its 35 kDa counterpart detected in *An. gambiae s.l.* SGEs. Interestingly, the 47 kDa band was uniquely observed in *An. albimanus*, and the 54 kDa antigenic band was faint but detected in all *Anopheles* antigenic profiles. All protein bands numbered in Figure 5C, corresponding to antigenic bands (Figure 5A and 5B), were submitted to MS analysis for identification. With the exception of protein bands numbered 1 (82 kDa, *An. gambiae*), 6 (30 kDa, *An. gambiae*), 8 (82 kDa, *An. arabiensis*), 13 (30 kDa, *An. arabiensis*), 14 (26 kDa, *An. arabiensis*), 15 (24 kDa, *An. arabiensis*),and 24 (54 kDa, *An. albimanus*) at least one protein was identified in all excised protein bands, resulting in the identification of 45 distinct proteins (17 housekeeping and 28 secreted proteins) according to their NCBI accession numbers (Additional file 4 and 5). As expected, several proteins could be identified in each excised band, and the same protein could also be identified in distinct excised bands from the same species as previously described [70]. Some proteins, such as the salivary apyrase [NCBI: gi|27372911] and anophensin [NCBI: gi|148189823] were identified in antigenic bands from all species belonging to the *Cellia* subgenus, including the non-African *An. stephensi* mosquito (Additional file 5). Orthologous proteins to *An. gambiae s.l*. antigens were identified in antigenic bands from the SGEs of *An. albimanus*, another non-African *Anopheles* mosquito (Figure 6). Interestingly, a GE-rich salivary gland protein [NCBI: gi|29501380] and a salivary gland protein [NCBI: gi|71389019] identified in *An. stephensi* and *An. albimanus*, respectively, shared 57.2% and 54.7% amino acid sequence identity with the anti-platelet protein [NCBI: gi|190576759] identified in the 30 kDa antigenic band from the *An. gambiae s.l.* complex (Additional file 6). These results highlight a potential link between protein sequence homology and the presence of cross-reactivity.

## Discussion

Reduction in exposure to malaria vectors either by controlling *Anopheles* density or avoiding their bites remains one of the most effective methods to protect human individuals from *Plasmodium* infections [15]. However, to determine the effectiveness of individual or collective anti-vector measures, it would be useful to develop new indicators that can measure the kinetic variations of individual exposure to specific malaria vectors within a population. By eliciting an antibody response linked to the level of exposure, antibody responses to salivary antigenic proteins from malaria vectors were proposed as valuable immunological markers to estimate host/vector contact [26,28-30,72]. Thus, the evaluation of the molecular diversity of salivary proteins from different *Anopheles* species can be used as a proxy to select genus-, subgenus- and species-specific salivary candidates for subsequent evaluation as immunological markers for *Anopheles* exposure [55]. The present study assessed the level of molecular and antigenic diversity and relatedness of secreted salivary proteins from major malaria vector species.

### Diversity of salivary protein among different species of the *Anopheles* genus

Vector species of the *Anopheles* genus throughout the world have different levels of phylogenetic relatedness [3]. Variation in the mitochondrial cytochrome oxidase subunit 2 (COII) sequence has been widely used to display the phylogenetic relationships and population structure of anopheline mosquitoes [73,74]. Molecular phylogenetic analysis using COII protein sequences from six selected malaria vectors (*An. gambiae*, *An. arabiensis*, *An. stephensi*, *An. funestus, An. albimanus* and *An. darlingi*) indicated that genetic distances between species were in agreement with their taxonomic classification. In addition, taking into account all of the secreted salivary proteins available from public databases for these six mosquito species, a pairwise protein-protein sequence analysis demonstrated that the proportion of salivary protein sequence homology decreases according to increasing phylogenetic distance between *Anopheles* species. Similar observations were reported for salivary proteins from phlebotomine sandflies [75], underlining this diversity of salivary secreted proteins inside a hematophagous arthropod genus. Comparative analyses of sialotranscriptomes between *Anopheles* species highlighted that secreted salivary proteins are less conserved than housekeeping proteins [48,49,52,57]. Heterogeneous secreted salivary protein repertoires among *Anopheles* species are consistent with the existence of secreted salivary proteins occurring throughout the *Anopheles* genus and others limited to the subgenus or species level.

### Genus-specific anopheline secreted proteins

Despite the low number of sequences available for some *Anopheles* species, several salivary protein families were found in all *Anopheles* species tested using agglomerative hierarchical clustering. These protein families included the apyrase/5’nucleotidase, antigen 5/gvag, GE-rich/30 kDa, long and short form D7, mucin/13.5 kDa, SG3, SG7, SG10 or hypothetical 6.2 kDa protein families. At the protein level, the clustering of salivary secreted proteins identified by MS confirmed that some proteins, including proteins from the apyrase/5’nucleotidase and 30 kDa/GE-rich/anti-platelet families, are present in all the investigated *Anopheles* species. Several of these protein families were also found in the saliva of other hematophagous arthropods, which could result from convergent evolution or a common ancestry [32,76]. For instance, members of the apyrase/5’nucleotidase and antigen 5/gvag protein families are found in the salivary glands of diverse hematophagous insect and tick species across the Arthropoda phylum. Interestingly, members from the GE-rich/30 kDa protein family are exclusively found in salivary glands of both culicine and anopheline female mosquitoes [49,57,77]. Here, other protein families appeared to be exclusively found in saliva from anopheline mosquitoes, such as SG3, SG7 or hypothetical 6.2 kDa proteins, offering the opportunity to use these proteins as genus-specific immunological markers. However, cross-reactivity is likely to occur when antigenic proteins share more than 70% amino acid identity [78]. Most of these orthologous protein sequences belonging to the *Anopheles* genus mostly shared less than 70% identity, minimising the probability of characterising conserved epitopes inside the *Anopheles* genus [43,79].

Although an antigenic band was commonly detected at 54 kDa in all *Anopheles* species, MS did not identify common or orthologous secreted protein in these antigenic bands that could explain the observed cross-reactivity. The low protein abundance in the corresponding gel bands and incomplete molecular sequencing data for some of the *Anopheles* species could explain this lack of identification. However, members of the GE-rich/30 kDa/anti-platelet family were identified in antigenic bands from the four *Anopheles* species, and are thus potential candidates to serve as pan-*Anopheles* genus markers of immunological exposure. Complementary experiments are required to evaluate the lack of cross-reactivity against salivary proteins from other mosquito species from the *Culicidae* family. Interestingly, in cases of human allergic reactions involving the production of IgE antibodies in response to mosquito bites, some salivary allergens from *Aedes aegypti* mosquito species have been characterized [34,80]. Among *Aedes aegypti* salivary allergens tested, recombinant forms of the 68 kDa salivary apyrase, the 37-kDa protein belonging to the D7 family and the 30 kDa salivary gland allergen were demonstrated to elicit an IgE responses in mosquito-allergic individuals. These data underline the antigenicity of some salivary proteins including 30 kDa family members, and the opportunity to use such proteins for the diagnosis and the desensitisation of mosquito allergic individuals.

### Subgenus-specific anopheline secreted salivary proteins

Combinations of subgenus-specific immunological markers may be an alternative for assessing exposure to *Anopheles* bites. Moreover, such markers could be helpful in identifying the anopheline subgenus involved in malaria transmission in places where several vector subgenera with different behaviour and vectorial capacity are encountered within the same area, notably in Asia [81,82]. *In silico* analysis of secreted salivary protein sequences from the six *Anopheles* species tested in the present study indicated that secreted salivary proteins sharing between 70% and 90% amino acid sequence identity are mainly clustered according to their subgenus affiliation. Thus, several salivary secreted proteins, such as TRIO, gSG2, gSG6 proteins and numerous hypothetical proteins with low molecular weights (Hypothetical 4.2/13, 8.2, 10, 12, or 15/17 kDa proteins), clustered only in the *Cellia* subgenus. The same observation was made for the *Nyssorhynchus* subgenus (*e.g.*, salivary peroxidase). Some of the secreted salivary proteins identified by MS were uniquely detected in the *Anopheles* species belonging to the *Cellia* subgenus, such as salivary apyrase, anophensin, D7 proteins or TRIO proteins. *In silico* analysis revealed that members of the gSG2 and gSG6 protein families are well conserved inside the *Cellia* subgenus with at least 67% and 77% identity, respectively, among *An. gambiae*, *An. funestus* and *An. stephensi*. Among these two protein families, *An. gambiae* gSG6, which is a reliable marker for exposure to *An. gambiae*[83-85] bites, was recently reported to be a good indicator for exposure to bites from three main African malaria vectors from *Cellia* sb. (*i.e*., *An. gambiae*, *An. arabiensis* and *An. funestus*). This cross-recognition could result from shared epitopes among these orthologous SG6 proteins [38,86]. According to their low molecular weights (approximately 11.7 and 13 kDa, respectively), gSG2 and gSG6 proteins should be contained in the large 11 kDa antigenic band detected in the *Cellia* subgenus. However, MS did not identify these last two proteins. Description of the salivary gland protein repertoire from *An. gambiae* performed by Kalume and colleagues identified gSG6 proteins only using a gel-free approach [87]. Thus, unsuccessful identification of these small proteins could be attributed to the combination of a high number of salivary proteins of this molecular weight and a low number of MS spectra generated by these small proteins, rendering this complex protein mixture unidentifiable by MS. The application of gel-free proteomic methods might increase salivary proteome coverage, especially concerning secreted protein with low molecular weights [88]. As orthologous secreted salivary proteins belonging to the same subgenus clustered only at important sequence identity levels, shared-epitopes are likely to occur, increasing the likelihood of observing cross-reactivity among these subgenus-specific proteins. Thus, identification of apyrase, anophensin and TRIO orthologs in antigenic bands from *An. gambiae s.l.* and *An. stephensi* SGEs highlight the potential of these salivary proteins to be *Cellia* subgenus-specific immunological markers.

### Species complex-specific anopheline secreted salivary proteins

The *Anopheles* subgenera encompass several groups of closely related species that are morphologically indistinguishable, such as the *An. gambiae s.l.* species complex, which includes at least 6 species [89-91]. *In silico* analysis revealed a high degree of homology (> 90%) among salivary protein sequences from each selected mosquito of this complex (*i.e.*, *An. gambiae* and *An. arabiensis*). Moreover, protein profiles and protein repertoires were highly similar between these two closely related *Anopheles* species, although one was collected in the field (*i.e.*, *An. arabiensis*) and the other came from continuous laboratory rearing (*i.e.*, *An. gambiae*). These results point to the likelihood that salivary protein candidates from either of these species would be able to assess exposure to *Anopheles* mosquitoes pertaining to the *An. gambiae s.l.* species complex. Using pooled sera from individuals mainly exposed to *An. gambiae, An. arabiensis* and *An. funestus*[71], most intense antigenic bands (40, 35 and 11 kDa) were revealed in SGEs from both species, suggesting that strong antibody responses against *An. gambiae s.l.* SGEs are elicited following exposure to these mosquitoes. Indeed, in a recent study conducted in the South of France, a positive association between mosquito exposure and the level of antibody response was reported and this immunological response appeared to be species-specific [92]. The major antigenic bands observed in *An. stephensi* and *An. albimanus* SGEs were about 4-fold less intense than the most intense antigenic bands detected in *An. gambiae s.l.* SGEs. Potential common antigenic salivary proteins were identified in major antigenic bands from *An. gambiae* and *An. arabiensis* salivary gland protein profiles, including anti-platelets, anophensin and proteins from the D7 and SG1 families. Even if members of these protein families are present throughout the *Cellia* subgenus, low amino acid sequence identities can occur between *An. gambiae s.l.* and other species, which could drastically affect antibody binding. Cross-reactivity usually implies a lower affinity for the cross-reactive antigen compared to the primary antigen [43]. The development of better quantitative methods, such as ELISA or Luminex®, combined with the production of salivary antigenic protein candidates may provide more distinct antibody responses to specific mosquito bites from the cross-reactivity attributed to partial shared-antigens.

### Species-specific anopheline secreted salivary proteins

It would be interesting to use immunological tools to assess individual exposure to a specific *Anopheles* species, even to closely related phylogenetically species, in areas harbouring several *Anopheles* species with distinct vector competences and behaviours. For instance, the *An. gambiae s.l.* complex encompasses species that often occur in sympatry but profoundly differ in their ability to transmit malaria parasites, in host feeding preferences, larval habitat requirements or responses to select vector control measures [90,93]. Thus, characterisation of *Anopheles* species-specific immunological markers may help to implement adapted vector-control strategies and assess their efficiency to prevent host/vector contact at the individual level. Additionally, the combination of different species-specific immunological markers using multiplex techniques, such as Luminex® [94], may increase sensitivity [38,95] and specificity of the test. Such multiplexing of validated antigenic salivary proteins will distinguish exposure to the bite of malaria vectors from that of non-vector *Anopheles*. Species-specific immunological markers may provide a more detailed view of the history of exposure at an individual level in retrospective studies. Thus, these markers may be useful to determine the implication of different vector species in malaria epidemics. *In silico* analysis indicated that 73 (27%) salivary proteins have no orthologous proteins in other *Anopheles* species at the lowest amino acid sequence identity threshold tested (40% identity). In addition, MS only identified 18 secreted in unique *Anopheles* species. Some of these species-specific salivary proteins, including mainly proteins with low molecular weights, are not found in SGEs from other hematophagous arthropod species (Additional file 2). Thus, these can serve as potential immunological markers for the assessment of individual exposure to specific *Anopheles* species.

Although *in silico* analysis, protein patterns and protein repertoires indicated a low diversity of secreted salivary proteins between *An. gambiae* and *An. arabiensis*, the detection of three antigenic bands exclusively in *An. arabiensis* SGEs suggest that closely related species could be distinct at the antigenic level. Unfortunately, MS analysis could not successfully identify the corresponding proteins from the gel bands. To identify these antigenic bands, a two-dimensional immunoproteomic approach using a fluorescence-based method could be an alternative [96]. Although the challenge appears more important for closely related mosquito species, our data suggest that identification of species-specific immunological markers seems reasonably conceivable. Moreover, this hypothesis is supported by our recent work on the existence of species-specific serological responses against *Ae. caspius* SGEs [92].

## Conclusions

The present study assessed, for the first time, the sialome diversity among different *Anopheles* species at the molecular and antigenic levels by combining *in silico*, proteomic and immuno-proteomic approaches. Our results demonstrate that salivary protein sequence identities among different species from one Culicidae genus are heterogeneous, with salivary proteins present throughout the *Anopheles* genus, or specific at the subgenus or species levels. This result demonstrates that salivary proteins from closely related species exhibit molecular diversity despite their common pharmacological activities (*e.g.* anti-haemostatic or immunomodulatory activities). This work supports the idea that genus-, subgenus- and species-specific salivary proteins can be used to develop immunological markers of individual exposure to malaria vectors. In complement to entomological methods, such immunological markers of exposure may be useful in the evaluation of anti-vector intervention strategies. In addition, development of species-specific immunological markers may help determine the implication of different vector species in malaria epidemics and provide further understanding of the vectorial system in a given area.

## Methods

### Sera samples

Sera from 5 individuals living in the Senegalese village of Dielmo (13°45’N, 16°25’W), sampled in March 1995 when malaria was holoendemic were used in this study [94]. These individuals were exposed to high levels of malaria transmission (about 200 infective bites/person/per year) and mosquito bites (human biting rate about 23.2), with *An. gambiae* (11%)*, An. arabiensis* (56%) and *An. funestus* (33%) as principal vectors [71]. These individuals did not travel outside Senegal country in the twelve months prior to blood sampling. The protocol was approved by the Senegal National Ethics Committee (Dakar, Senegal). The informed consent of each participant was obtained at the beginning of the study, after a thorough explanation of its purpose.

### Phylogenetic analysis

The cytochrome oxidase II (COII) protein sequences from *An. gambiae*, *An. arabiensis*, *An. stephensi*, *An. albimanus, An. funestus, An. darlingi* and *Aedes aegypti* were retrieved from NCBInr database (May 10^th^, 2011) and multiple sequence alignment was performed with Clustal W 1.7 multiple sequence alignment program [97] which is included in Molecular Evolutionary genetic Analysis 5 (MEGA 5) programs package [62]. The evolutionary history was inferred using the Neighbour-Joining method in MEGA 5 [98]. The *Ae. aegypti* sequence was taken as an outgroup. The bootstrap consensus tree inferred from 10,000 replicates [99] is taken to represent the evolutionary history of the taxa analyzed. Branches corresponding to partitions reproduced in less than 50% bootstrap replicates are collapsed. The percentage of replicate trees in which the associated taxa clustered together in the bootstrap test (10,000 replicates) are shown next to the branches [99]. The evolutionary distances were computed using the Poisson correction method and are in the units of the number of amino acid substitutions per site. All positions containing gaps and missing data were eliminated. Evolutionary analyses were conducted in MEGA5 [62]. Protein sequence alignments were generated in Geneious Pro 5.6.4 http://www.geneious.com/.

### *In silico* analysis

#### Sequences retrieval and pairwise protein sequence comparison

All salivary protein sequences were retrieved in FASTA format from the online non-redundant National Center for Biotechnology Information protein database (NCBInr, NIH, Bethesda, June 15^th^, 2011) under the taxonomies *An. gambiae* [7165], *An. arabiensis* [7173], *An. stephensi* [30069], *An. funestus* [62324], *An. darlingi* [43151] and *An. albimanus* [7167], using the search term “salivary” in any fields of their description text. Signal peptides were predicted by submission of the protein sequences to the SignalP server 3.0 [64], allowing the determination of putative secreted proteins. Putative secreted proteins sequences from each *Anopheles* species were blasted again each other using “BLAST 2 Sequences” [63] with default parameters (NCBI, National Library of Medicine, http://blast.ncbi.nlm.nih.gov/Blast.cgi). For each protein from species A, the raw BLAST score and percentage identity of the best match with species B was manually selected according to the E-value. Duplicates of protein sequences from species B that matched several proteins in species A were then removed according to their lowest E-value in order to select unique and best homologous protein sequence between two species. The BLAST Score Ratio approach was adopted to represent similarities between salivary proteins pertaining to different *Anopheles* species [66,67]. In this approach, the raw BLAST score resulting from a comparison between a query saliva protein sequence from species A and a protein sequence from species B is divided by the self-BLAST score obtained with the BLAST of the query protein sequence from species A with itself. The resulting normalized BLAST score vary from 0 (no match) to 1 (perfect match). The use of such normalized scores overcomes several problems associated with the use of E-values, such as biases entailed in comparisons among different databases, falsely high E-values assigned to low-complexity proteins and falsely low E-values based on small regions of high similarities [66,67]. The average normalized BLAST score as well as average percentage identity between salivary proteins from two species were then calculated.

#### Sequences clustering

All putative secreted sequences were merged in a single FASTA file and submitted to the CD-HIT server [68] for hierarchical clustering (H-CD-HIT) as describe elsewhere [69]. Briefly, the program performs clustering three times in succession with decreasing similarity thresholds. First, clustering start with the input dataset at a high identity threshold (≥ 90%). The longest sequence becomes the representative of the first cluster. Then, each remaining sequence is compared to the representatives of all existing clusters. If the predefined similarity threshold is met, the sequence is grouped into the most similar cluster. Otherwise, a new cluster is defined with that sequence as the representative. The last two steps of the hierarchical clustering (≥ 70% and ≥ 40% similarity threshold) start with representatives of the previous clustering runs and the whole process produces a hierarchical structure. The percentage identities are calculated by counting the numbers of identical amino acids between two protein sequences by using a short word filter (For details see the user’s guide at the following web link (http://www.bioinformatics.org/cd-hit/cd-hit-user-guide.pdf). Each salivary protein sequence was further blasted against a database containing SGP sequences identified so far in 26 hematophagous arthropod species (*i.e., An. gambiae* [7165], *An. arabiensis* [7173], *An. stephensi* [30069], *An. funestus* [62324], *An. albimanus* [7167], *An. darlingi* [43151], *Ae. aegypti* [7159], *Ae. albopictus* [7160], *Ochlerotatus triseriatus* [7162], *Culex tarsalis* [7177], *Cx quinquefasciatus* [7176], *Triatoma brasiliensis* [65344], *T. infestans* [30076], *Rhodnius prolixus* [13249], *Cimex lectularius* [79782], *Glossina morsitans morsitans* [37546], *Xenopsylla cheopis* [163159], *Stomoxys calcitrans* [35570], *Simulium vittatum* [7192], *S. nigrimanum* [683695], *Ornithodoros parkeri* [140564]*, O. coriaceus* [92741], *Argas monolakensis* [34602], *Ixodes pacificus* [29930], *I. scapularis* [6945] and *Rhipicephalus sanguineus* [34632]) by sialotranscriptomic studies [100]. Proteins matching with an E-value < 1×10^-10^ were considered putative homologs [66].

### Mosquitoes and salivary gland extraction

Uninfected 5-day-old adult females of the *An. gambiae s.s.*, *An. arabiensis*, *An. stephensi* and *An. albimanus* species were used in this study. *An. stephensi* and *An. albimanus* species were reared at the Institut Pasteur (CEntre de Production et Infection des *Anopheles,* CEPIA, Paris). *An. gambiae* was reared at the Institut de Recherche pour le Développement (laboratoire de Lutte contre les Insectes Nuisibles**,** Montpellier). *An. arabiensis* species was collected on the field at the larvae stage at Dakar (Senegal) at the end of the rainy season in September 2008 [101] and identified by polymerase chain reaction (PCR) [102]. After emergence, adult mosquitoes were reared at the Institut de Recherche pour le Développement (UR24, Dakar). All mosquitoes used in the experiments were maintained under identical standard conditions: 26°C and 60% humidity, took no blood meals and were maintained on a diet of 10% syrup solution. The salivary glands from adult mosquito females were dissected under a stereomicroscope at 4X magnification as previously described [92]. The salivary glands from each experiment were pooled by strains into a microcentrifuge tube on ice in phosphate-buffered saline (PBS) and then stored frozen at −20°C until needed.

### Sample preparation

Salivary glands were disrupted by ultrasonication (Vibracell 72412, Bioblock Scientific, Illkirch, France) for 5 min on ice at maximum amplitude. Salivary gland homogenates were then centrifuged for 15 min at 16,100 ×g [103] and protein concentration of the supernatant was determined in duplicate by the Lowry method (DC Protein assay Kit, Bio-Rad) according to the manufacturer’s instructions. Salivary gland proteins were then concentrated by precipitation with acetone (Sigma, St Louis, MI), and were suspended in a UTC buffer containing 8M urea (Sigma), 2M thiourea (Sigma), 4% (w/v) CHAPS (Sigma) and 30 mM Tris (Sigma), adjusted to pH 8.5 in order to obtain a protein concentration adjusted to 2.5μg/μL.

### One-dimensional electrophoresis (SDS-PAGE)

For each species, 20 μg of salivary gland proteins were separated onto a 12% SDS-PAGE in a PROTEAN II xi or Mini (BioRad, Hercules, USA). A broad range molecular weight marker (BioRad) was loaded on each gel. Gels were stained with Sypro Ruby (BioRad) according to the manufacturer’s protocol and digitalized using the Typhoon^TM^ Trio Image scanner (GE Healthcare, UK). Salivary glands densitometry profiles were analyzed using the ImageQuant^TM^ TL software (GE Healthcare, UK). Background subtraction was performed and the densitometry profiles were normalized to take into account global differences [54].

### Immuno-blotting

For each species, 15 μg of SGPs were separated onto a 12% SDS-PAGE as described above. SGPs were minimally labelled with CyDye 5 according to the manufacturer’s protocol (GE Healthcare) prior electrophoresis, as described elsewhere [96]. Gels were then transferred to a nitrocellulose membrane (0.45-μm, Amersham Pharmacia, Saclay, France) by semidry blotting (0.8 mA per cm^2^) [96]. Blots were saturated 1 hour at room temperature with 5% w/v non-fat dried milk, and were carried out with human sera diluted at 1/50 in phosphate buffer saline (PBS) containing 0.1% v/v tween-20 with 5% w/v non-fat dried milk. After an overnight incubation, blots were incubated with anti-human Fcγ/IgG FITC conjugated antibody 1/1000 (Sigma, St Louis, MI). Immunoblots were directly digitalized using a Typhoon^TM^ Trio Image scanner (GE Healthcare) and densitometric analysis of IgG immune profiles was performed using ImageQuant^TM^ TL software (GE Healthcare), as previously described [54].

### Protein band excision and in-gel digestion

Each loading tracks were excised covering the totality of the each lane as previously described [54,68], using Shimadzu Biotech Xcise System (Champs sur Marne, France). Salivary protein identification was made in duplicate on two distinct gels. Protein bands were digested overnight at 37°C with sequencing-grade trypsin (12.5 μg/mL; Promega Madison, WI, USA) in 50 mM NH_4_HCO_3_ (Sigma). The resulting peptides were extracted with 25 mM NH_4_HCO_3_ for 15 min, dehydrated with acetonitrile (ACN) (Sigma), incubated with 5% acid formic (Sigma) for 15 min under agitation, then dehydrated with ACN, and finally completely dried using a SpeedVac. Samples were then stored at −20°C before analysis by mass spectrometry (MS).

### Mass spectrometry analysis

For MS analysis, a LCQ DecaXPplus (ThermoFinnigan, San Jose, CA) ion trap was used. Nano-liquid separation of peptides was carried out using an Ettan MDLC chromatographic system (GE Healthcare) in high throughput configuration. Ten microliters of the digest were first trapped on a zorbax 300SB-C18 5 × 0.3 mm column and eluted at a flow rate of approximately 200 nl/min on a zorbax 300SB-C18, 3.5 μm, 150 × 0.075 mm by a linear gradient of eluant B (0.1% Formic acid, 84% ACN) in eluant A (1% Formic acid). The chromatographic system was piloted by the Unicorn 5.01 software (GE Healthcare). MS measurements were done on a LCQTM Deca XP Plus ion trap mass spectrometer (ThermoFinnigan) equipped with a LCQTM nanospray ionization source. A spray voltage of 1.8 kV was applied to the liquid junction via an in-union high voltage contact coupled to a silicaTip emitter (New Objective). Operation of the mass spectrometer was fully automated during the entire procedure using the Excalibur 1.3 data system (ThermoFinnigan). Continuous cycles of one full scan (m/z 500 to 1700) followed by three data-dependent MS/MS measurements at 35% normalized collision energy were done. MS/MS measurements were allowed for the three most intense precursor ions with a maximum rejection time limit of 1 min.

### MS data analysis

All MS/MS spectra from one species were gathered and sequence database searched using the Bioworks 3.1 (ThermoFinnigan) or Mascot Daemon v.2.2.2 (Matrix Science, London, UK). The MS/MS spectra were searched against the non-redundant protein database (NCBInr, NIH, Bethesda) of *An. gambiae* [7165]*, An. arabiensis* [7173]*, An. stephensi* [30069] and *An. albimanus* [7167] together (July 27th, 2009, 16677 sequences). The following search parameters were used: precursor-ion mass tolerance of 0.8 Da, fragment ion tolerance of 0.8 Da with methionine oxidation and cysteine carboxyamidomethylation specified as differential modifications, and a maximum of one missed cleavage site allowed. Scaffold (version Scaffold_3.6.2, Proteome Software Inc., Portland, OR) was used to validate MS/MS based peptide and protein identifications. Peptide identifications were accepted if they could be established at greater than 95.0% probability as specified by the Peptide Prophet algorithm [104]. Protein identifications were accepted if they could be established at greater than 95.0% probability and contained at least 1 identified peptide. Protein probabilities were assigned by the Protein Prophet algorithm [105].

## Competing interests

The authors declared no conflict of interest concerning the work in this paper.

## Authors’ contributions

Conceived and designed the experiments: AL, FA, RC. Performed the experiments: FA, AL, BE. Analyzed the data: FA, AL, BuS. Contributed reagents/materials/analysis tools: BrS, PM, VC, GS, FT. Wrote the paper: FA, AL. All authors read and approved the final manuscript.

## Supplementary Material

Additional file 1 **Average percentage identity between local alignments of secreted salivary proteins from six *Anopheles*species.** All secreted salivary protein sequences from each *Anopheles* species *(An. gambiae, An. arabiensis, An. stephensi, An. funestus, An. albimanus* and *An. darlingi)* matching at least one other salivary protein in another species at 40% identity threshold (*q.v.,* Additional file 2) were recovered and blasted again each other. The percentage identity of the best match (lowest E-value) was recovered with the protein NCBInr accession number and normalized BLAST scores were calculated based on raw BLAST scores and raw self-BLAST scores. When a unique salivary protein from target species B matched several proteins in reference species A, only the best match (lowest E-value) was selected. Average normalized BLAST scores ± SD and percentage identities are indicated in bold and summarized on Figure 1C. Click here for file

Additional file 2 **Hierarchical clustering of secreted salivary gland proteins from six *Anopheles*species.** A three step clustering was performed at ≥ 90%, ≥ 70% and ≥ 40% identity threshold with the H-CD-HIT server on secreted salivary proteins from *An. gambiae, An. arabiensis, An. stephensi, An. funestus, An. albimanus and An. darlingi.* Clusters are sorted into protein families. The NCBI accession number is indicated for each protein. * indicate the representative (i.e., longest) protein sequence of each cluster. The percentage identity between the representative protein sequence (*) and other protein sequences is given for each cluster. Protein in bold are new clusterised proteins at each identity threshold. Results from this table are graphically represented on Figure 2. Click here for file

Additional file 3 **Proteins identified by MS in salivary gland extracts of four *Anopheles*species.** All MS/MS spectra resulting to every protein bands from each species *(An. gambiae*, *An. arabiensis*, *An. stephensi* and *An. albimanus)* were gathered and searched on sequence databases of the four *Anopheles* species together. A list of all unique proteins identified in salivary gland extracts in both replicates is presented for each *Anopheles* species. Salivary gland proteins were sorted according to their signal peptide prediction (SignalP Neural Network) [63,65] to discriminate secreted proteins from housekeeping ones. Click here for file

Additional file 4 **Hierarchical clustering of putative secreted proteins identified in *Anopheles*salivary gland extracts.** Proteins from *An. gambiae*, *An. arabiensis*, *An. stephensi* and *An. albimanus* SGEs were identified by mass spectrometry after in-gel trypsin digestion. Protein sequences were submitted to SignalP 3.0 server [65] to select putative secreted proteins and were hierarchically clustered at ≥ 90%, ≥ 70% and ≥ 40% identity threshold with CD-HIT web server [68]. * indicate the representative (*i.e.*, longest) protein sequence of each cluster. *Anopheles* species in which secreted salivary proteins were identified are indicated. The last common taxon encompassing homologous proteins at the genus level is indicated according to *in silico* results (*q.v.* Additional file 2). n.a.: non-available (*i.e.*, uncharacterized protein sequences that were not recovered in the *in silico* analysis). Lines in bold indicate proteins identified in antigenic bands (Figure 5A, Additional file 5). AGA, *An. gambiae*; AGA^#^, *An. gambiae* PEST strain (Pink Eye STandard); AAR, *An. arabiensis*; AST, *An. stephensi*; AAL, *An. albimanus*; MW: Molecular weight. Click here for file

Additional file 5 **Proteins identified by MS in antigenic protein bands from salivary gland extracts from *Anopheles*species.** Salivary gland proteins identified in each antigenic protein band from *An. gambiae*, *An. arabiensis*, *An. stephensi* and *An. albimanus SGEs* are indicated. Band numbers correspond to those indicated on Figure 5C. Click here for file

Additional file 6 **Alignment of members of the GE-rich/30 kDa/anti-platelet protein family from *An. gambiae*, *An. stephensi* and *An. albimanus*.** The numbers in the sequence titles indicate the NCBI accession number. Click here for file
