# Supplementary material for: Anopheles salivary gland proteomes from major malaria vectors
Source: BMC Genomics. 2012 Nov 13;13:614. doi: 10.1186/1471-2164-13-614 (PMC3542285; doi:10.1186/1471-2164-13-614)
Supplement: Additional file 4 — Hierarchical clustering of putative secreted proteins identified in Anophelessalivary gland extracts. Proteins from An. gambiae, An. arabiensis, An. stephensi and An. albimanus SGEs were identified by mass spectrometry after in-gel trypsin digestion. Protein sequences were submitted to SignalP 3.0 server [65] to select putative secreted proteins and were hierarchically clustered at ≥ 90%, ≥ 70% and ≥ 40% identity threshold with CD-HIT web server [68]. * indicate the representative (i.e., longest) protein sequence of each cluster. Anopheles species in which secreted salivary proteins were identified are indicated. The last common taxon encompassing homologous proteins at the genus level is indicated according to in silico results (q.v. Additional file 2). n.a.: non-available (i.e., uncharacterized protein sequences that were not recovered in the in silico analysis). Lines in bold indicate proteins identified in antigenic bands (Figure 5A, Additional file 5). AGA, An. gambiae; AGA#, An. gambiae PEST strain (Pink Eye STandard); AAR, An. arabiensis; AST, An. stephensi; AAL, An. albimanus; MW: Molecular weight. [file 1471-2164-13-614-S4.doc]

|  | % identity | | |  |  |  |  |  | Protein identification probabilities | | | | Specificity |
| --- | --- | --- | --- | --- | --- | --- | --- | --- | --- | --- | --- | --- | --- |
|  | ≥ 90% | ≥ 70% | ≥ 40% | Accession no. | Protein description | Prot. tax. | length | MW (kDa) | AGA | AAR | AST | AAL | (genus level) |
| Cl. 0 |  |  |  |  |  |  |  |  |  |  |  |  |  |
| 0 | * | * | 60% | **gi|4538887** | **D7-related 1 protein** | ***AGA*** | **165aa** | **19** | **100%** | **100%** | **100%** |  | ***Cellia*** |
| 1 | 98% | 98% | 60% | gi|16225961 | short form D7r1 salivary protein | *AAR* | 165aa | 19 | 100% | 100% | 100% |  | *Cellia* |
| 2 | 98% | 98% | 60% | gi|58389659 | AGAP008284-PA | *AGA^#^* | 165aa | 19 | 100% | 100% | 100% |  | *n.a* |
| 3 | * | * | 45 | gi|16225977 | short form D7clu4 salivary protein | *AST* | 164aa | 18 |  |  | 99% |  | *Cellia* |
| 4 | * | * | * | gi|16225980 | short form D7clu5 salivary protein | *AST* | 166aa | 19 |  |  | 100% |  | *Cellia* |
| 5 | 96% | 96% | 96% | gi|21314941 | contact-activation-inhibitor protein hamadarin | *AST* | 166aa | 19 |  |  | 100% |  | *Cellia* |
| 6 | * | * | 41% | gi|31222496 | AGAP008281-PA | *AGA^#^* | 165aa | 19 | 98% | 100% |  |  | *n.a* |
| Cl. 1 |  |  |  |  |  |  |  |  |  |  |  |  |  |
| 0 | * | * | 60% | **gi|15718081** | **D7 protein** | ***AST*** | **315aa** | **36** |  | **100%** | **100%** |  | ***Anopheles*** |
| 1 | * | * | 55% | **gi|16225958** | **long form D7 salivary protein** | ***AAR*** | **311aa** | **36** | **99%** |  |  |  | ***Anopheles*** |
| 2 | 96% | 96% | 55% | gi|31222545 | AGAP008278-PA | *AGA^#^* | 311aa | 36 | 99% |  |  |  | *n.a* |
| 3 | * | * | * | **gi|158296846** | **AGAP008279-PA** | ***AGA^#^*** | **321aa** | **37** | **100%** | **100%** |  |  | ***n.a*** |
| Cl. 2 |  |  |  |  |  |  |  |  |  |  |  |  |  |
| 0 | * | * | * | **gi|27372911** | **salivary apyrase** | ***AST*** | **575aa** | **64** | **100%** | **100%** | **100%** | **95%** | ***Anopheles*** |
| 1 | * | 78% | 78% | **gi|58377530** | **AGAP011026-PA** | ***AGA^#^*** | **570aa** | **63** | **100%** | **100%** |  |  | ***n.a*** |
| 2 | * | * | 46% | gi|58394160 | AGAP011971-PA | *AGA^#^* | 558aa | 62 | 100% | 100% |  | 95% | *n.a* |
| Cl. 3 |  |  |  |  |  |  |  |  |  |  |  |  |  |
| 0 | * | * | * | **gi|13537664** | **gSG1b protein** | ***AGA*** | **385aa** | **44** | **100%** | **100%** | **100%** |  | ***Cellia*** |
| 1 | 99% | 99% | 99% | gi|58380426 | AGAP000548-PA | *AGA^#^* | 385aa | 44 | 100% | 100% |  |  | *n.a.* |
| 2 | * | * | 56% | gi|27372941 | putative salivary protein SG1C | *AST* | 383aa | 44 |  |  | 95% |  | *Cellia* |
| Cl. 4 |  |  |  |  |  |  |  |  |  |  |  |  |  |
| 0 | * | * | * | gi|37201975 | GE rich salivary gland protein | *AST* | 269aa | 28 |  |  | 100% |  | *Anopheles* |
| 1 | * | * | 48% | **gi|71389019** | **salivary gland protein** | ***AAL*** | **240aa** | **26** |  |  |  | **99%** | ***Anopheles*** |
| 2 | * | * | 62% | **gi|190576759** | **anti-platelet protein** | ***AGA*** | **252aa** | **27** | **100%** | **100%** |  |  | ***Anopheles*** |
| Cl. 5 |  |  |  |  |  |  |  |  |  |  |  |  |  |
| 0 | * | * | * | gi|27372903 | putative 53.7 kDa salivary protein | *AST* | 516aa | 56 |  |  | 100% |  | *An. stephensi* |
| 1 | * | 70% | 70% | gi|118787092 | AGAP005822-PA | *AGA^#^* | 513aa | 55 |  | 100% |  |  | *n.a.* |
| Cl. 6 |  |  |  |  |  |  |  |  |  |  |  |  |  |
| 0 | * | * | * | **gi|18873404** | **hypothetical protein** | ***AGA*** | **401aa** | **47** | **99%** | **100%** | **100%** |  | ***Cellia*** |
| 1 | 99% | 99% | 99% | gi|31203047 | AGAP000609-PA | *AGA^#^* | 401aa | 46 | 99% | 100% |  |  | *n.a.* |
| Cl. 7 |  |  |  |  |  |  |  |  |  |  |  |  |  |
| 0 | * | * | * | gi|18389917 | TRIO protein | *AGA* | 391aa | 44 | 100% |  |  |  | *Cellia* |
| 1 | 97% | 97% | 97% | **gi|58396245** | **AGAP001374-PA** | ***AGA^#^*** | **391aa** | **44** | **100%** | **100%** |  |  | ***n.a.*** |
| Cl. 8 |  |  |  |  |  |  |  |  |  |  |  |  |  |
| 0 | * | * | * | **gi|34556108** | **putative gVAG protein precursor** | ***AGA*** | **260aa** | **30** | **100%** | **100%** |  |  | ***Anopheles*** |
| 1 | * | 84% | 84% | **gi|27372895** | **salivary antigen-5 related protein** | ***AST*** | **259aa** | **29** |  |  | **100%** |  | ***Anopheles*** |
| Cl. 9 | * |  |  | gi|347965870 | AGAP001424-PA | *AGA^#^* | 800aa | 21 |  |  |  |  | *n.a.* |
| Cl. 10 | * |  |  | **gi|118783568** | **AGAP004192-PA** | ***AGA^#^*** | **659aa** | **73** |  | **100%** | **100%** | **100%** | ***n.a.*** |
| Cl. 11 | * |  |  | **gi|118793578** | **AGAP002102-PA** | ***AGA^#^*** | **593aa** | **67** | **100%** | **100%** | **100%** |  | ***n.a.*** |
| Cl. 12 | * |  |  | **gi|4539761** | **salivary peroxidase** | ***AAL*** | **591aa** | **65** |  |  |  | **100%** | ***Nyssorhynchus*** |
| Cl. 13 | * |  |  | **gi|118778070** | **AGAP007393-PB** | ***AGA^#^*** | **488aa** | **54** | **100%** | **100%** | **100%** |  | ***n.a.*** |
| Cl. 14 | * |  |  | **gi|158300147** | **AGAP012407-PA** | ***AGA^#^*** | **472aa** | **53** | **100%** | **100%** | **100%** | **100%** | ***n.a.*** |
| Cl. 15 | * |  |  | **gi|347964149** | **AGAP000610-PA** | ***AGA^#^*** | **431aa** | **47** |  |  |  |  | ***n.a.*** |
| Cl. 16 | * |  |  | **gi|4210615** | **SG1 protein** | ***AGA*** | **401aa** | **47** | **100%** | **100%** |  |  | ***An. gambiae*** |
| Cl. 17 | * |  |  | **gi|30267888** | **secretion protein gp65** | ***AAL*** | **386aa** | **43** |  |  |  | **100%** | ***Nyssorhynchus*** |
| Cl. 18 | * |  |  | gi|158287728 | AGAP011024-PA | *AGA^#^* | 262aa | 30 |  |  |  | 95% | *n.a.* |
| Cl. 19 | * |  |  | **gi|29501376** | **short D7-4 salivary protein precursor** | ***AST*** | **168aa** | **18** |  |  | **100%** |  | ***Anopheles*** |
| Cl. 20 | * |  |  | gi|118788764 | AGAP008478-PA | *AGA^#^* | 161aa | 17 |  | 95% |  |  | *n.a.* |
| Cl. 21 | * |  |  | **gi|148189823** | **anophensin** | ***AST*** | **142aa** | **16** | **95%** | **98%** | **100%** |  | ***Anopheles*** |
| Cl. 22 | * |  |  | gi|4127309 | hypothetical protein | *AGA* | 122aa | 13 |  | 99% |  |  | *An. gambiae* |
