# Supplementary figures and images for: Anopheles salivary gland proteomes from major malaria vectors
Source: BMC Genomics. 2012 Nov 13;13:614. doi: 10.1186/1471-2164-13-614 (PMC3542285; doi:10.1186/1471-2164-13-614)

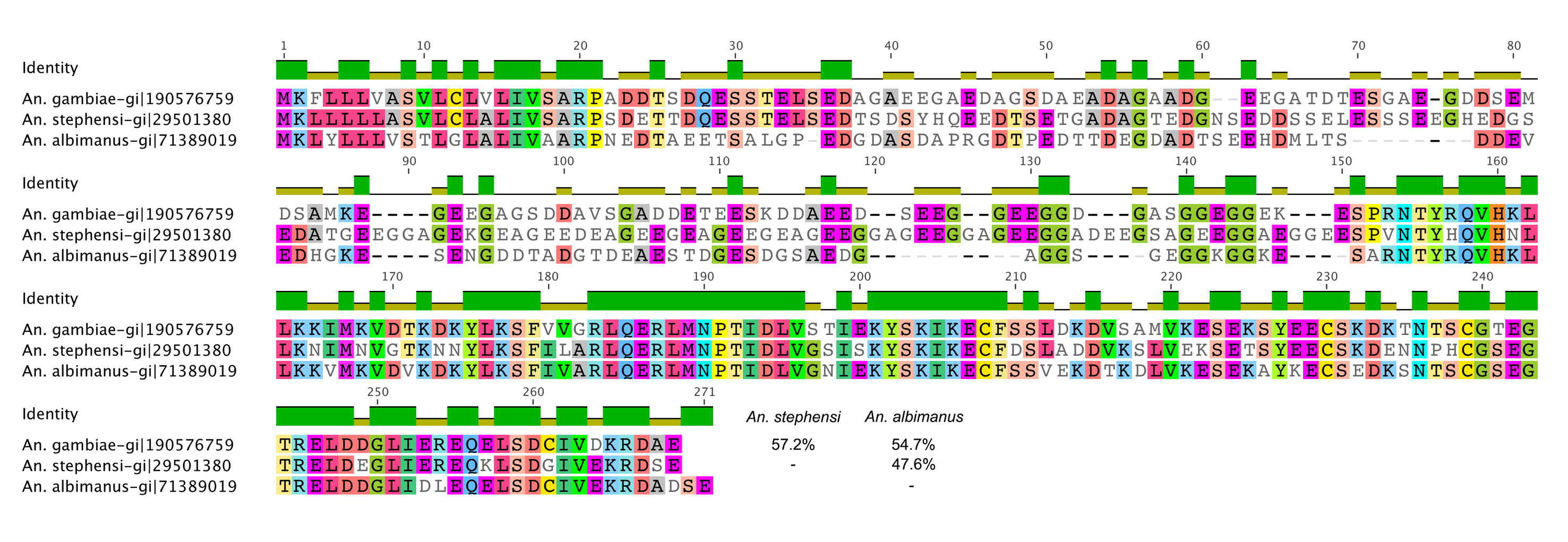

Supplement: Additional file 6 — Alignment of members of the GE-rich/30 kDa/anti-platelet protein family from An. gambiae, An. stephensi and An. albimanus. The numbers in the sequence titles indicate the NCBI accession number. [file 1471-2164-13-614-S6.png]
